# Advanced Management of Open Apex Utilizing Platelet-Rich Fibrin and Bone Graft As Apical Barriers With Mineral Trioxide Aggregate (MTA) Obturation: A Detailed Case Report

**DOI:** 10.7759/cureus.60883

**Published:** 2024-05-22

**Authors:** Yash Sinha, Akansha Tilokani, Prasanti K Pradhan, Neelanjana Majee, Bhushan Wandile

**Affiliations:** 1 Department of Conservative Dentistry and Endodontics, Kalinga Institute of Dental Sciences, KIIT Deemed to be University, Bhubaneswar, IND; 2 Department of Medicine, Jawaharlal Nehru Medical College, Datta Meghe Institute of Higher Education and Research, Wardha, IND

**Keywords:** endodontic treatment, mineral trioxide aggregate (mta), demineralized bone matrix (dmbm), platelet-rich fibrin (prf), apexification, open apex

## Abstract

Management of open apex cases in endodontics poses a significant challenge, especially in immature teeth with necrotic pulps. Traditional apexification techniques have been the mainstay of treatment, aiming to induce the formation of a calcific barrier at the root apex. However, newer approaches incorporating biological materials such as platelet-rich fibrin (PRF) and demineralized bone matrix (DMBM) have emerged as promising alternatives. This article presents a case report of an 18-year-old male patient who presented with fractured upper central incisors, with the upper right central incisor displaying an open apex due to trauma sustained eight years prior. The treatment plan involved apexification using a combination of DMBM and PRF, with mineral trioxide aggregate (MTA) utilized as an apical barrier. The procedure was performed under rubber dam isolation, meticulously removing necrotic pulp tissue, irrigating with sodium hypochlorite solution, and placing a calcium hydroxide medicament. Subsequent visits included the placement of DMBM and PRF mixture into the canal space to create an apical barrier, followed by MTA placement and final restoration. Follow-up examinations at 3 and 12 months revealed the tooth to be asymptomatic and functionally normal, with radiographic evidence of osseous repair and complete apical closure. This case underscores the efficacy of a multimodal approach utilizing DMBM, PRF, and MTA in successfully managing open apex cases. Further research and long-term follow-up studies are warranted to validate this treatment modality's predictability and long-term success.

## Introduction

Traumatic dental injuries, such as crown fractures and apex disruption, pose significant challenges in endodontic management, particularly when dealing with immature teeth. Open apex cases often result from traumatic incidents and present complexities in root canal treatment due to the absence of a mature apical barrier. Traditional apexification techniques involving the induction of a calcific barrier at the root apex have shown limited success rates and may require extended treatment periods [1]. There has been growing interest in using biologically based materials to promote tissue regeneration and apex closure in open apex cases. Platelet-rich fibrin (PRF) and demineralized bone matrix (DMBM) have emerged as promising adjuncts in endodontic therapy due to their osteogenic and wound-healing properties [2,3].

PRF, a concentration of platelets and growth factors derived from the patient's blood, has been shown to enhance tissue healing and promote angiogenesis and cell proliferation [4]. Similarly, DMBM, composed of osteoinductive proteins and growth factors, is a scaffold for new bone formation and facilitates tissue regeneration [5]. Mineral trioxide aggregate (MTA) has gained widespread acceptance as an apical barrier material due to its excellent sealing ability, biocompatibility, and favorable outcomes in endodontic therapy [6]. MTA promotes mineralized tissue formation and facilitates open apices' closure, improving clinical outcomes and long-term success rates [7].

Combining these biological materials, including PRF, DMBM, and MTA, offers a comprehensive approach to apexification in open cases. The synergistic effects of these materials promote tissue regeneration, enhance healing, and facilitate the formation of a functional apical barrier, leading to improved clinical outcomes and predictable treatment results [8]. In this case report, we present the successful management of an open apex using a combination of PRF and DMBM as an apical barrier, with MTA as a sealing material. The treatment aims to promote tissue regeneration and facilitate apex closure, restoring tooth function and integrity.

## Case presentation

**Figure 1:**
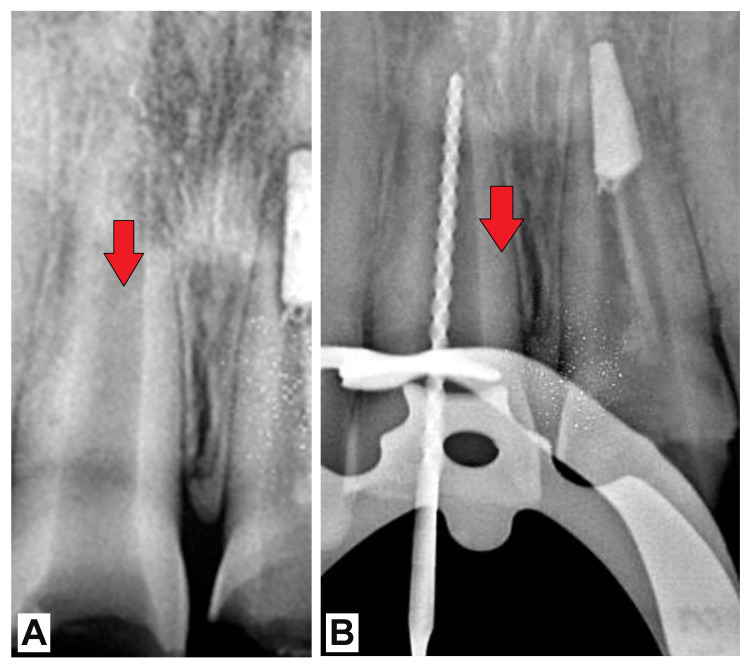
(A) Preoperative radiograph of the patient and (B) working length (red arrows)

An 18-year-old male patient sought treatment at the Department of Endodontics with complaints of fractured upper central incisors on both the right and left sides. The patient reported a history of trauma to the same teeth approximately eight years ago, resulting from a fall. Upon clinical examination, Ellis class III fractures were noted in both central incisors. Additionally, the upper right central incisor exhibited an open apex. Electric pulp testing revealed nonvitality in the affected tooth. Following thorough evaluation and assessment, a treatment plan was formulated to address the open apex and restore the functionality of the affected tooth. The plan involved a multistep approach aimed at apexification, utilizing a combination of DMBM, PRF, and MTA. Under strict rubber dam isolation, access to the pulp chamber was prepared, and the working length was determined radiographically (Figure 1). Necrotic pulp tissue was delicately removed using circumferential filing techniques, accompanied by frequent irrigation with 2.5% sodium hypochlorite (NaOCl) solution. Following meticulous cleaning and drying of the canal, a calcium hydroxide medicament was applied, and the tooth was temporarily restored.

**Figure 2:**
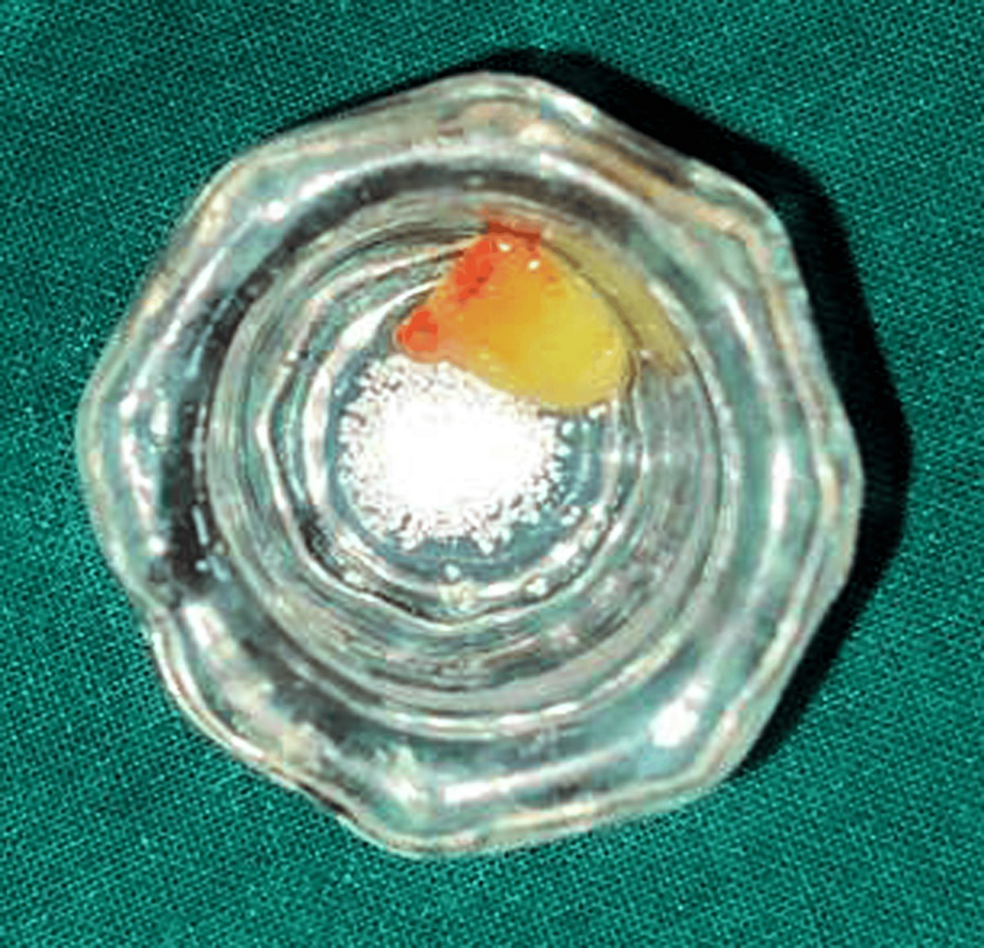
Decalcified freeze-dried bone graft with PRF PRF: platelet-rich fibrin

The canal medicament was removed during the subsequent appointment, and the canal was irrigated with NaOCl. A decalcified freeze-dried bone allograft (DMBM) and PRF were carefully placed into the canal to create an apical barrier (Figure 2).

**Figure 3:**
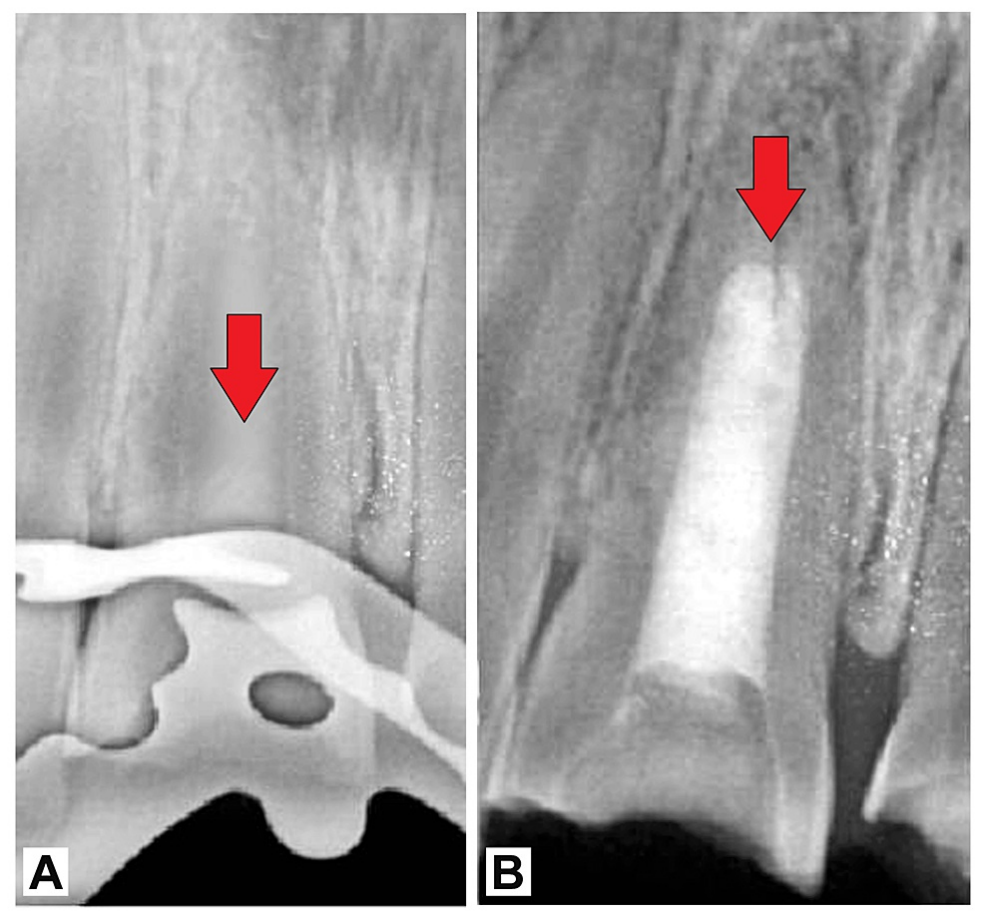
(A) Four-millimeter apical plug formed using decalcified freeze-dried bone graft with PRF and (B) entire canal filled with MTA until canal orifice (red arrows)

Radiographic confirmation of a 4-mm thick apical plug was obtained, ensuring adequate sealing and barrier formation (Figure 3). Subsequently, MTA was placed in the canal space to seal the apex further and promote tissue regeneration. The tooth was then temporarily restored and assessed the following day to confirm the setting of MTA. Final restoration with composite resin was performed to restore both function and aesthetics.

**Figure 4:**
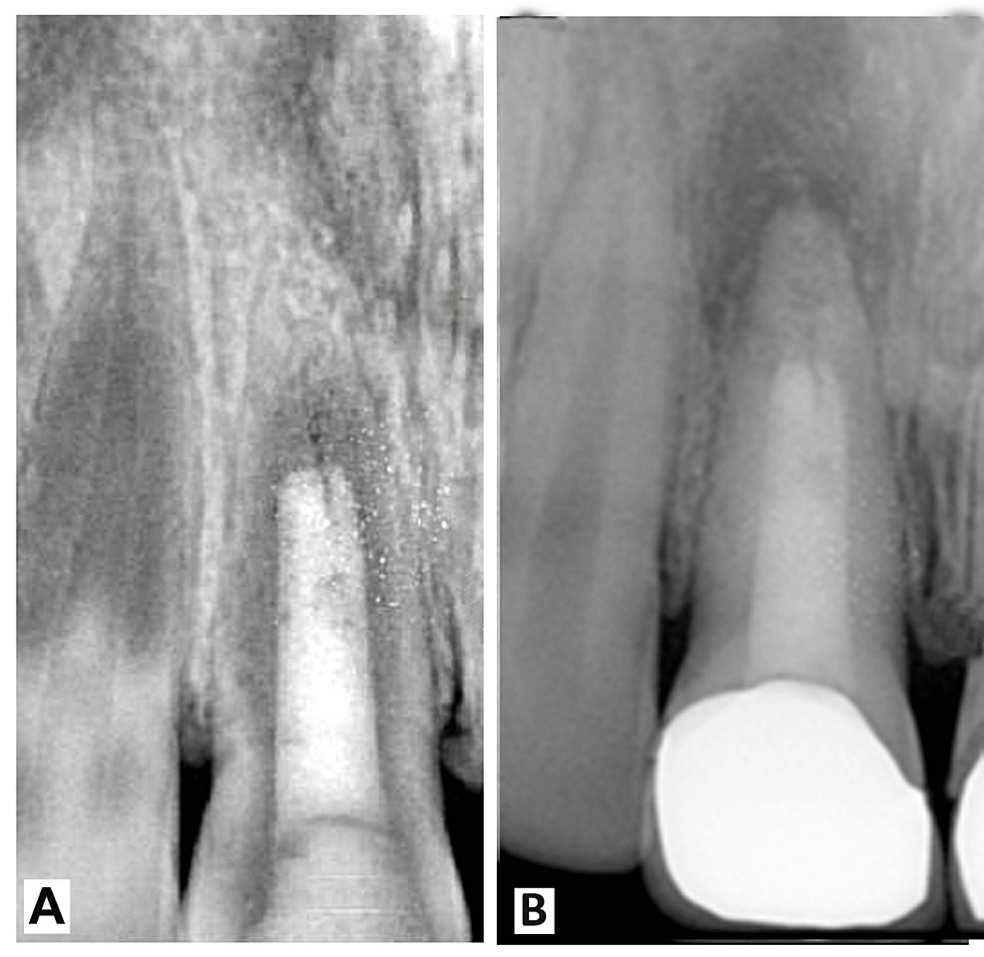
(A,B) Follow-up images of the patient

Follow-up examinations at 3 and 12 months after treatment revealed the tooth to be asymptomatic and functionally normal. Radiographic evaluation demonstrated osseous repair and complete apical closure, confirming the success of the treatment approach in managing the open apex and restoring the health and integrity of the affected tooth (Figure 4).

## Discussion

Managing open apex cases presents unique challenges in endodontic therapy, particularly when dealing with immature teeth with necrotic pulps. Traditional apexification techniques involve the induction of a calcific barrier at the root apex to facilitate obturation. However, newer approaches incorporating biological materials such as PRF and DMBM have shown promising results in promoting tissue regeneration and apex closure [9]. In the present case, a combination of DMBM, PRF, and MTA was utilized to achieve successful apexification and closure of the open apex. DMBM serves as a scaffold for tissue regeneration, providing a framework for depositing new hard tissue. PRF, derived from the patient's blood, contains a high concentration of growth factors that promote wound healing and tissue regeneration. These biological materials have been shown to enhance the formation of a functional apical barrier and facilitate the regeneration of periapical tissues [10,11].

The use of MTA as an apical barrier further enhances the success of the treatment. MTA is widely recognized for its biocompatibility, sealing ability, and ability to stimulate hard-tissue formation [12]. It forms a tight seal with dentin, preventing microleakage and bacterial ingress into the root canal system. Moreover, MTA has been shown to induce the formation of a calcific barrier at the apex, leading to successful apexification and root canal obturation [13]. The successful outcome of the present case is consistent with previous studies reporting the efficacy of DMBM, PRF, and MTA in the management of open apex cases. A systematic review by Guerrero et al. evaluated the clinical and radiographic outcomes of apexification with various materials and reported favorable outcomes using MTA in conjunction with biological materials such as PRF and DMBM [14]. Similarly, a study by Lv et al. demonstrated successful apexification and apical closure in immature teeth with necrotic pulps using a combination of MTA and PRF [15]. However, it is important to note that this treatment approach's long-term success and predictability require further investigation. Long-term follow-up studies are needed to assess the stability of the apical barrier, the integrity of the root canal filling, and the health of periapical tissues over time. Additionally, larger scale clinical trials comparing different treatment modalities are warranted to determine the optimal approach for managing open apex cases.

## Conclusions

In conclusion, the successful management of the open apex case using a combination of DMBM, PRF, and MTA underscores the potential of this multimodal approach in endodontic therapy. By promoting tissue regeneration and providing a biocompatible apical barrier, DMBM and PRF contributed to the successful closure of the apex, while MTA ensured the effective sealing of the root canal system. While the outcomes of this case are promising, further research and long-term follow-up studies are necessary to validate the efficacy and predictability of this treatment modality. Nevertheless, the present case highlights the importance of employing innovative techniques and biological materials to address challenging endodontic cases and achieve favorable patient outcomes.
